# HIV-2 as a model to identify a functional HIV cure

**DOI:** 10.1186/s12981-019-0239-x

**Published:** 2019-09-05

**Authors:** Joakim Esbjörnsson, Marianne Jansson, Sanne Jespersen, Fredrik Månsson, Bo L. Hønge, Jacob Lindman, Candida Medina, Zacarias J. da Silva, Hans Norrgren, Patrik Medstrand, Sarah L. Rowland-Jones, Christian Wejse

**Affiliations:** 1 Department of Translational Medicine, Lund University, Malmö, Sweden; 2 Nuffield Department Medicine, University of Oxford, Oxford, UK; 3 Department of Laboratory Medicine, Lund University, Malmö, Sweden; 4 Department of Infectious Diseases, Aarhus University Hospital, Aarhus, Denmark; 5 Bandim Health Project, Indepth Network, Bissau, Guinea-Bissau; 6 Department of Clinical Sciences Lund, Lund University, Lund, Sweden; 7 National HIV Programme, Ministry of Health, Bissau, Guinea-Bissau; 8 National Public Health Laboratory, Bissau, Guinea-Bissau; 9 GloHAU, Center for Global Health, Department of Public Health, Aarhus University, Aarhus, Denmark; 10 Systems Virology, Department of Translational Medicine, Lund University, BMC B13, 221 84 Lund, Sweden

**Keywords:** HIV-1, HIV-2, Dual-infection, Functional cure, Disease progression, West Africa

## Abstract

Two HIV virus types exist: HIV-1 is pandemic and aggressive, whereas HIV-2 is confined mainly to West Africa and less pathogenic. Despite the fact that it has been almost 40 years since the discovery of AIDS, there is still no cure or vaccine against HIV. Consequently, the concepts of functional vaccines and cures that aim to limit HIV disease progression and spread by persistent control of viral replication without life-long treatment have been suggested as more feasible options to control the HIV pandemic. To identify virus-host mechanisms that could be targeted for functional cure development, researchers have focused on a small fraction of HIV-1 infected individuals that control their infection spontaneously, so-called elite controllers. However, these efforts have not been able to unravel the key mechanisms of the infection control. This is partly due to lack in statistical power since only 0.15% of HIV-1 infected individuals are natural elite controllers. The proportion of long-term viral control is larger in HIV-2 infection compared with HIV-1 infection. We therefore present the idea of using HIV-2 as a model for finding a functional cure against HIV. Understanding the key differences between HIV-1 and HIV-2 infections, and the cross-reactive effects in HIV-1/HIV-2 dual-infection could provide novel insights in developing functional HIV cures and vaccines.

## HIV-1 and HIV-2 epidemiology and pathogenesis

HIV-2 was first discovered in 1986 in West Africa [1]. Although HIV-2 has been found in other parts of Africa, Europe, India and the United States; West Africa has consistently had the largest HIV-2 prevalence [2–5]. In 1994, the first studies of HIV-2 reported a reduced rate of disease progression compared with HIV-1 among female sex workers in Senegal [6]. In 1997, it was reported that HIV-2 infected individuals had twice as high mortality compared with HIV negative individuals in Guinea-Bissau [7]. This was confirmed in later studies that showed mortality rates two to fivefold higher in HIV-2 infected individuals compared with HIV-negative individuals in Guinea-Bissau [8, 9]. Other studies, from The Gambia and France, compared HIV-1 and HIV-2 infection and reported a generally slower CD4+ T-cell decline in HIV-2 infected individuals [10, 11]. HIV-2 infected individuals therefore exhibit longer asymptomatic stages than HIV-1 infected individuals [12]. However, in individuals reaching AIDS, HIV-1 and HIV-2 share a similar clinical spectrum, with the exception of a lower incidence of Kaposi’s sarcoma in HIV-2 infected individuals [13, 14]. Interestingly, studies have also reported that similar baseline viral load and CD4+ T-cell levels predict similar prognosis for HIV-1 and HIV-2 infected individuals [15, 16]. This could indicate that disease prognosis is determined during the early stages of both types of HIV infections.

The viral set-point has been suggested to be 10–28 fold lower in HIV-2, with lower levels of viraemia persisting into clinical stages of disease [17, 18]. As a consequence, HIV-2 infection is characterized by lower transmission rates at both the horizontal and vertical levels [19, 20]. Moreover, AIDS seem to occur at a relatively lower viral load level in HIV-2 compared with HIV-1 infection, although the CD4 count is often higher in HIV-2-infected subjects when AIDS-defining illnesses develop [12, 14, 21]. The lower transmission rates of HIV-2 compared with HIV-1 is further highlighted by the parallel HIV-1 increase and HIV-2 decline seen in West Africa between 1990 and 2010 [2, 4, 5, 22].

Several reports have indicated that only approximately 15–25% of HIV-2 infected individuals will progress to AIDS if following a natural course of disease [3, 23, 24]. However, these assumptions were based on data from HIV-2 infected individuals without information on infection date. On the one hand, lack of infection date will inevitably select for individuals with a slower disease progression rate than the average. On the other hand, such population will also enter the study at a more advanced stage. These biases will create a contradiction that will be difficult to adjust for when estimating true disease progression rates. In 2018, data from individuals with an estimated date of infection showed that the disease trajectory was almost identical between HIV-1 and HIV-2 infections, albeit at approximately half the rate among HIV-2 infected individuals [12, 25, 26]. Importantly, this showed that AIDS will develop among the majority of HIV-2 infected individuals without antiretroviral treatment (ART). Nevertheless, although no such indication was seen in the study, the existence of a subset of HIV-2-infected subjects who maintain long-term viral control and have a normal life expectancy cannot be entirely excluded since this would require a complete follow-up to the end-stage (AIDS or death) of all study participants [27]. However, in such subgroup, the time to AIDS would be longer than the predicted human lifespan, meaning that the age at HIV-2 infection would be a determining factor for the size of the group. In fact, the median age at infection was 38 years in the HIV-2 infected group [27]. This, together with the lack of information on infection date, could explain previous results of the high proportion of HIV-2 infected individuals not developing HIV-related disease.

## HIV-1 and HIV-2 virology and immunology

The HIV-1 and HIV-2 epidemics constitute multiple different introductions of simian immunodeficiency viruses (SIV) into the human population [28]. HIV-1 has its origin from SIV of the chimpanzee, whereas HIV-2 originated from the SIV of the sooty mangabey [29, 30]. Due to the parallel evolution of SIV and HIV in simian and human populations there is a distinct genetic diversity between HIV-1 and HIV-2. To date, a large number of groups, subtypes, subsubtypes and circulating recombinant forms have been described for HIV-1, and at least nine groups of HIV-2 have been described (termed A–I) [28]. Group A and B are most common in HIV-2 infection, although intergroup recombinants between group A and B has been described [30]. However, and despite their different origins, HIV-1 and HIV-2 are related retroviruses and show approximately 55% similarity in Gag and Pol, and 35% similarity in Env on the protein level (the overall similarity level is approximately 55% on the nucleotide level) [31]. Although the virus types share transmission routes and target cells, contrasting results in terms of replicative fitness and cytopathicity have been reported [32, 33].

It is well established that blood plasma viral load is lower in HIV-2 compared with HIV-1 infection [34]. It would therefore make sense that viral replication could largely explain the difference in pathogenicity between the two viruses. Studies of natural disease progression caused by HIV-1 have indicated large variations in viral loads between individuals and the difference in plasma viral load may not fully explain the difference in rate of disease progression between the two virus infections. Interestingly, a recent study showed that CD4+ T-cell levels during the asymptomatic stage of infection was stronger associated with HIV-2 disease progression rate than with CD4+ T-cell decline [35]. Further studies are therefore needed to determine the causative effects and predictive values of viral load and CD4+ T-cell levels in natural disease progression of both HIV-1 and HIV-2 infection [36, 37].

It has been suggested that untreated HIV-1 and HIV-2 infected individuals with similar CD4+ T-cell levels have similar levels of *gag* mRNA transcripts, indicating that substantial viral transcription occurs in HIV-2 infected individuals despite the generally lower viral loads [38]. Interestingly, the *tat*/*gag* ratio between HIV-1 and HIV-2 infections has been shown to differ [39–41]. Altogether, these studies suggest that *tat* mRNA levels are reduced compared with *gag* mRNA levels in cells from untreated HIV-2 infected individuals, whereas *tat* mRNA levels are more abundant than *gag* mRNA levels in cells from HIV-1 infected individuals. Since *tat* mRNA represents early transcripts, these results could reflect a reduced rate of recent cell infections in HIV-2 infection. It is also possible that post-transcriptional control of viral production could be involved in differences in HIV-1 and HIV-2 pathogenesis [42].

Lower virus production in HIV-2 compared with HIV-1 infection may also reflect a lower activation state in infected cells, or that HIV-2 is less responsive to activation. The long terminal repeat (LTR) of both HIV-1 and HIV-2 regulates the expression of the virus in response to cellular transcription signals. The HIV-2 LTR differs from HIV-1 in numbers and type of transcription binding elements and enhancers, leading to reduced responsiveness to transcription factors present in activated T-cells [43]. It has been shown that the HIV-2 LTR does not respond as well as the HIV-1 LTR to tumour necrosis factor alpha [44]. Similar results were obtained in experiments measuring viral replication [45]. There could also be differences in activation of HIV-1 and HIV-2 infected cells. In contrast to HIV-1, the HIV-2 envelope glycoprotein was found to stimulate production of higher levels of gamma interferon and interleukin 16 (both inhibit viral replication), and lower levels of interleukin 4 (stimulates viral replication) [46]. Further studies may result in novel molecular targets for functional HIV cure strategies.

**Fig. 1:**
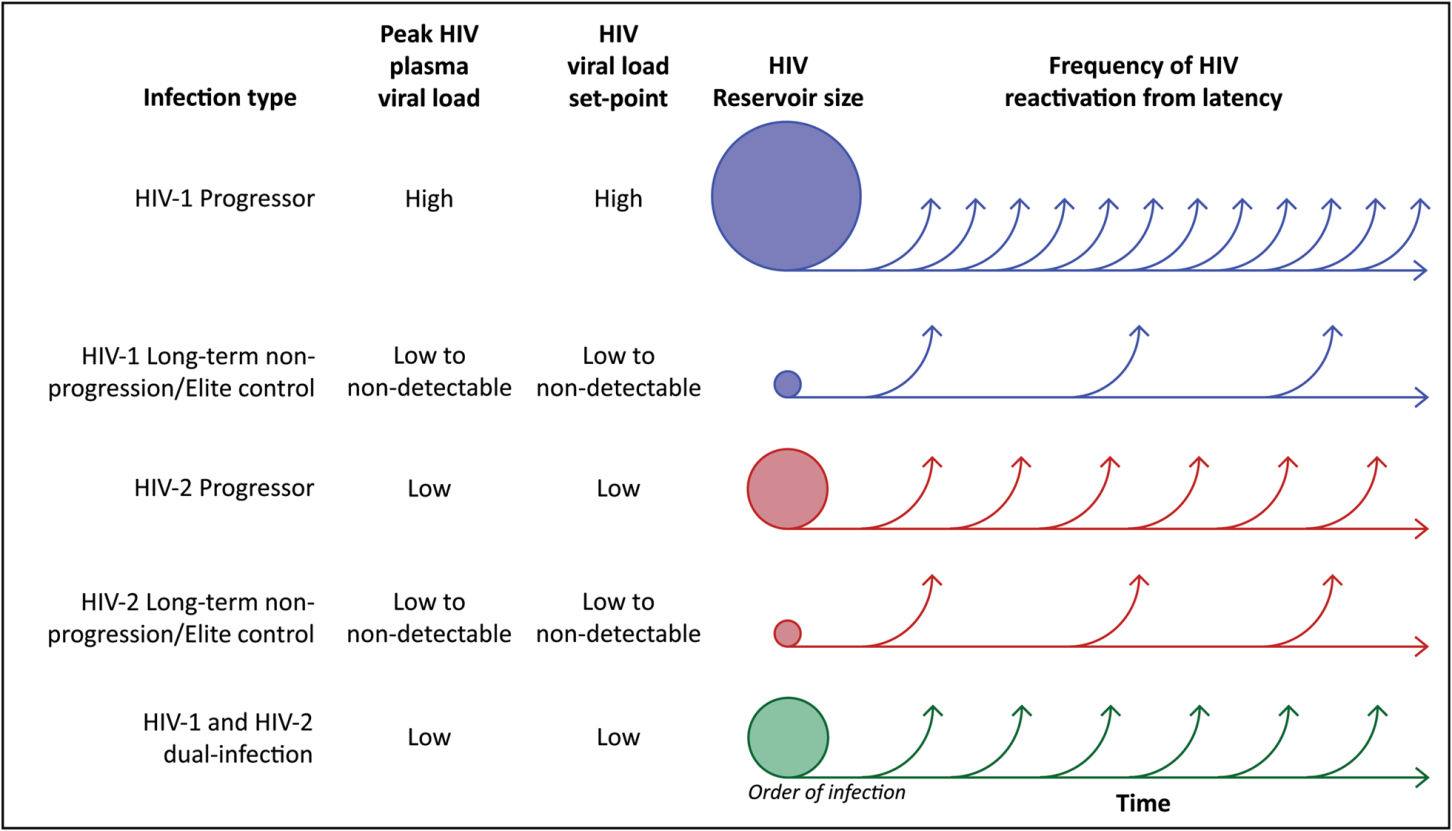
Schematic of potential differences between HIV progressor groups in frequency of HIV reactivation from latency. Clearance or control of the latent HIV reservoir remains one of the main obstacles to achieve a functional HIV cure. Although the viral reservoir in HIV-1 infection has been extensively studied, much less is known about the reservoir size or reactivation frequency from this reservoir in HIV-2, and HIV-1 and HIV-2 dual-infection. This figure outlines possible differences in HIV reservoir size and reactivation frequency between the main HIV infection types and progressor groups discussed in this review. The importance of the order of infection in HIV-1 and HIV-2 dual-infection has been highlighted in the figure, and it is likely that the HIV reservoir size and reactivation frequency will differ between depending of the order of HIV infections types

The latent HIV-1 reservoir has been studied extensively, and the HIV-1 reservoir establishment is associated with the seeding of virus during the acute HIV-1 infection before the adaptive arm of the immune system starts to partially control the virus replication [47, 48]. It is also well established that HIV-1 remain quiescent in long-lived CD4+ memory T-cells. Moreover, viral rebound is normally seen only a few weeks after secession of ART even in patients with previous long-term virus suppression. Hence, virus latency in these cells remain one of the main challenges for finding a functional cure against HIV. The size of the virus reservoir has been measured using different protocols, including qPCR of cell-bound virus DNA and mRNA, quantification of ex vivo reactivation of virus mRNA and proteins, as well as in ex vivo virus outgrowth assays. In HIV-1 long-term non-progressors (LTNP) and elite controllers (EC), that have been suggested as models for functional cure, the reservoir of latently infected cells is reduced compared with HIV-1 viraemic and treated individuals [49, 50]. Interestingly, conflicting results about proviral DNA levels in HIV-2 compared with HIV-1 infection have been reported. Two studies indicated similar proviral levels after adjusting for disease stage [51, 52]; whereas a study by Gueudin et al. [40] reported the opposite. Thus, further studies are needed to establish proviral DNA loads at different disease stages of HIV-2 infection, and how they differ from HIV-1 infection. Moreover, even though quantification of virus DNA by qPCR correlate with the size of the latent HIV-1 reservoir, these assays often overestimate the size of the replication competent latent HIV-1 reservoir. Although few studies have characterized the HIV-2 reservoir, it was recently reported that HIV-2 DNA could be quantified in transitional-memory cells from four of 14 ART naive HIV-2-infected individuals, and in central-memory cells from one of 14 ART naive HIV-2-infected individuals [41]. Approximately 100 HIV-2 DNA copies/10^6^ cells were detected in each of the specific memory cell subsets, respectively. However, HIV-2 in vitro reactivation was only observed in cells from three of the 14 individuals, suggesting presence of defective proviruses. In line with this, the predominance of defective proviral DNA in HIV-2 infected individuals on successful ART has recently been reported from studies of three virally suppressed individuals [53]. In this study, most of the HIV-2 genomes had large deletions, whereas hypermutations were noted in a smaller fraction of the sequences. However, complete understanding of the HIV-2 reservoir will require larger studies and the use of different protocols. Moreover, both in vitro and ex vivo studies of latency reversal agents used in HIV-1 clinical settings are needed for HIV-2 (reviewed in [54]). Still, from available proviral load data and studies of HIV-1 in LTNP and EC (that in many ways resemble HIV-2) it is plausible that the remission of HIV-2 would be relapse-free or delayed, and less frequent compared with the general HIV-1 case (Fig. 1).

Explanations for lower virus loads and reduced pathogenicity in HIV-2 compared with HIV-1 infections have also been attributed to different types of virus-controlling immune responses. Robust, broadly cross-reactive and polyfunctional virus-specific responses of both CD4+ and CD8+ T-cells have been shown to distinguish HIV-2 from HIV-1 infections [55–63]. In particular, HIV-2 Gag-specific T-cell responses have been shown to correlate with virus control [59, 61, 62]. Similarly, CD8+ T-cells co-expressing CD28 have been associated with controlled HIV-2 infection [18, 64]. Strikingly, when subjects from the “Caió cohort” with high and undetectable viral loads were compared, the main distinguishing feature was CD8+ T-cell responses to Gag, which were absent in 52% of viraemic participants (the “Caió cohort” was a large community-based cohort from the small rural village Caió, Guinea-Bissau) [63]. Gag-specific responses in HIV-2-infected subjects often show unusually high functional avidity, with the capacity to respond to picomolar quantities of antigen, and are at an earlier stage of differentiation than HIV-1-specific CD8+ T-cells, presumably retaining their ability to proliferate [58, 63]. In line with these observations, HIV-2-specific T-cells from LTNPs in the French cohort showed potent suppression of viral replication, in many cases exceeding the suppressive abilities of HIV-1-specific T-cells from elite controllers [64]. Given that high potency HIV-specific cytotoxic T-lymphocytes (CTL) without features of exhaustion and broad cross-reactivity seem to be required for killing of the latent reservoir, there are good arguments to suggest that CTL from HIV-2 infected viral controllers would meet these requirements [47].

The cellular involvement in modulation of HIV-2 infections within West African populations also comes from HLA associations with viral control (HLA-B*58:01, HLA-DPB1*10:01 and HLA-DRB1*11:01) or disease progression (HLA-B*15:03 and HLA-B*35) [65–67]. Furthermore, the function of natural killer (NK) cells appears more well-preserved during asymptomatic HIV-2 compared with HIV-1 infection, whereas the functionality of these cells seem to drop to levels found during HIV-1 infection with declining CD4+ T-cells [68]. Similarly to HIV-1 infection, chronic immune activation is linked to immunopathogenesis and progressive disease in HIV-2 infection [18, 69–75]. Elevated frequencies of T-cells expressing markers of activation and exhaustion appear to distinguish HIV-2 infected individuals that progress despite no or low level viraemia, which could be the consequence of long infection duration and/or virus replication not mirrored by plasma RNA levels [18, 70–72]. Although these findings could suggest that aviraemic HIV-2 infected individuals should be offered ART, there remains a group of aviraemic individuals with HIV-2 infection without evidence of immune activation who may not necessarily benefit from therapy [70].

Both potent and broadly neutralizing antibodies have been detected at different disease stages in HIV-2 infection [51, 76–79]. Moreover, although susceptibility of HIV-2 to neutralizing antibodies seem to vary according to the infecting virus strain, it is in general significantly elevated compared to HIV-1 [77–80]. Furthermore, mutants escaping from neutralizing antibodies seem to emerge less frequently in HIV-2 infection and do not reach fixation [79, 81, 82]. Fc-mediated effector functions, such as the effect of complement on antibody antiviral activity, have also been reported to be potent in HIV-2 infection [76]. In addition, antibodies with a broad ability to mediate antibody dependent cellular cytotoxicity (ADCC), and even cross-react with HIV-1, are found in HIV-2 infected individuals [83, 84]. Thus, despite low-level viraemia, strong antibody responses in HIV-2 infection are sustained during both asymptomatic and progressive HIV-2 infections and do not distinguish between patient groups in different disease stages [79]. The impact of antibodies on disease progression during HIV-2 chronic infection is, therefore, not entirely clear.

Finally, animal models for HIV analysis of virus transmission and AIDS development are rare, and chimpanzees are the only non-human primates that are susceptible to HIV-1 infection. However, due to ethics, risk of extinction, and an infection that rarely results in progressive disease, these animals are not used as an infection model for HIV [85]. Experimental HIV-2 infection models have instead been established using rhesus and pigtail macaques. Still, these infections mainly result in low or non-pathogenic infections [86, 87]. In contrast, serial in vivo passages of HIV-2 in baboons have resulted in the development of an AIDS-like disease model [88, 89]. It has also been reported that HIV-2 infection of humanized mice results in persistent viraemia and CD4+ T-cell loss [90]. These could therefore represent alternative models for testing efficacy of antiretroviral and latency reversal strategies. As for in vitro models, competition assays between HIV-1 and HIV-2 have demonstrated that the replication fitness of most HIV-2 isolates was lower and outcompeted by HIV-1 isolates [32], and others have shown that HIV-2 isolates from aviraemic individuals have reduced in vitro replication capacity compared to HIV-2 isolated during viraemia [91].

## HIV-1 and HIV-2 dual-infection

Dual-infection with HIV-1 and HIV-2 has been reported with a prevalence of up to 3.2% in West Africa [4, 92]. However, cross-reactivity in antibody testing and limited molecular testing to distinguish dual-infections have hampered the accuracy of prevalence estimates. Intriguingly, a possible protective effect of HIV-2 on subsequent incident HIV-1 infection was reported in 1995, and several studies reported that HIV-2 could alter HIV-1 infectivity and replication in vitro [93–95]. Moreover, HIV-2 infection has been shown to inhibit immunosuppression and simian AIDS after subsequent challenge with pathogenic SIV or SHIV in the Macaque model [96, 97]. In 2012, it was shown that HIV-2 could inhibit HIV-1 disease progression also in humans, resulting in almost twice as long time to AIDS and mortality among HIV-1 and HIV-2 dual-infected individuals compared with HIV-1 single-infected individuals [98, 99]. Importantly, the results showed that the slower disease progression was determined during the establishment of infection, and that the inhibitory effect was strongest among study participants where the HIV-2 infection preceded the HIV-1 infection (indicating the importance of the order of infections, Fig. 1). Moreover, in-depth analyses of CD4+ T-cell counts and HIV-1 diversity evolution indicated that the main difference between single and dual-infected individuals was determined during early HIV-1 infection. In support, results from the Bissau HIV cohort showed that the median CD4+ T-cell count was higher and the mortality lower in dual-infected individuals compared with HIV-1 single-infected individuals [100]. In contrast, a meta-analysis by Prince et al. [101] did not show any difference between HIV-1 single, and HIV-1 and HIV-2 dual-infected individuals. However, the data used was extracted from studies that were not designed for comparing survival between single and dual-infected individuals, and lacked information on estimated infection date and infection order among the dual-infected individuals. Most studies suffered from short periods of patient follow-up or observation time, and some were based on hospitalized patients with severe disease already at enrollment. Altogether, if identified, the determinants of the inhibition and slower disease progression in HIV-1 and HIV-2 dual-infection compared with HIV-1 single-infection could represent novel targets suitable for HIV cure strategies or vaccines. Moreover, it is possible that the viral reservoir in HIV-1 and HIV-2 dual-infected individuals is reduced compared to that in the majority of HIV-1 single-infected individuals. Cure strategies in dual-infected individuals may therefore be more successful compared with HIV-1 single-infected individuals (Fig. 1).

## Current functional cure strategies and the possibility of such studies in HIV-2 endemic areas

As the research field of HIV cure has matured over recent years, it has been necessary to define different concepts of the term ‘cure’ [48]. Within HIV-1 cure research, the aspect of ‘functional cure’ has emerged, or lately ‘relapse-free remission’ to define sustained suppression of virus without the need for ART. This means that a ‘functional cure’ does not have to result in complete absence of HIV in the body. Several different cure strategies have been suggested, e.g. stimulation of the latently infected cells to reduce the reservoir size; gene therapy to reduce the number of target cells; and immunotherapy to ameliorate the HIV-specific immune response [102–105]. An example of naturally occurring ‘functional cure’ are so-called elite controllers, which has been described in a small minority of HIV-1 patients [106–108]. Interestingly, this phenomenon seems to be much more frequent among HIV-2 infected individuals, and it is largely unknown why this is the case [27]. Therefore, there are important lessons to be learned from HIV-2 pathogenesis, and HIV-2 may represent a model to study relapse-free remission and open up new avenues towards how to induce relapse-free remission in HIV-1 infection (Fig. 1).

Antiretroviral treatment effectively suppresses, but does not cure HIV infection. Multiple therapeutic strategies have been pursued in HIV cure research, but there has been a particular focus on using latency-reversing-agents (LRAs) such as histone deacetylase inhibitors (HDACi), disulfiram, Protein C kinase agonists and Toll-like Receptor agonists, to activate HIV-expression in latently infected cells, and thereby exposing their infected status to the immune system and potentially facilitating immune or virus-mediated cell lysis [109]. This is usually termed “shock-and-kill” [103]. Yet, although clinical trials of many of these compounds have demonstrated that HIV latency can be disrupted in individuals on suppressive ART, this does not lead to a reduction in the frequency of latently infected cells or delayed viral rebound during analytical interruption of ART [110]. More recently, attempts to reverse latency with compounds that both activate virus and modulate immunity to enhance clearance of infected cells (so-called immunomodulatory LRAs), have been made [89]. The inability of latency-reversal interventions to impact the latent HIV reservoir in clinical trials has increased the scientific focus on immune-enhancement strategies towards a concept of relapse-free remission after cessation of therapy. This is also supported by the demonstration that even in individuals with no or extremely low levels of HIV in cells or plasma, there is rebound viraemia when ART is stopped [111, 112]. This emphasizes that an effective strategy to achieve long-term ART-free remission should include both a component that reduces the amount of HIV that persists on ART, and a component that improves anti-HIV immune surveillance of residual viruses. This could involve immune-based therapies with immune checkpoint inhibitors, TLR agonists, or HIV-specific broadly neutralising antibodies, which are currently under intense investigation for application in HIV prevention, treatment and cure [105, 113–115]. Additionally, starting ART shortly after infection has been a focus area, as this is associated with both a lower frequency of latently infected CD4+ T-cells in blood and tissue, and a better preserved T-cell function [116, 117]. Furthermore, early ART increases the likelihood of post-treatment control, i.e. the ability to achieve durable remission after interruption of ART, which was started in primary infection—a phenomenon initially described in the French VISCONTI cohort [118].

Despite recent disappointments on the possibility of LRAs leading to longstanding remission in HIV-1, there could be a case for trying LRAs and “shock-and-kill” therapies in HIV-2, since it may be a less fit and more sensitive virus. HIV-2 cytotoxic CD8+ T-cell responses and possibly antibody responses, either broadly neutralizing or mediating ADCC, may partly explain the delayed progression of HIV-1 in patients firstly infected with HIV-2 and later superinfected with HIV-1 [56, 59, 77–79, 84, 98, 99, 119–123]. Hence, if HIV-2 immune responses play a role in controlling the rate of HIV-1 disease progression in individuals with dual-infection, it is plausible that boosting immunity may be able to induce relapse-free remission in HIV-2. There is a need to elicit studies on remission-strategies among HIV-2 infected individuals as these studies hold important promises for achieving an increased understanding of how to achieve remission in HIV-1.

An important question is if there is sufficient capacity to undertake a functional cure study based on HIV-2 in the field sites where there is a sufficient number of relevant HIV-2 cases. There are undoubtedly numerous factors to take into consideration before embarking on such highly complex clinical trials, and a number of relevant concerns need to be taken into account (summarised in Table 1). Yet, these are all manageable challenges that have been overcome in previous studies (Table 2). For example, both the Bissau HIV cohort and the Guinea-Bissau police cohort have built a high standard trial capacity during the last decades. Moreover, a therapeutic HIV-1 vaccine trial has already been completed in the Bissau HIV cohort [124, 125]. Despite limited settings, large HIV treatment trials such as the PIONA trial have been possible through an experienced clinical trial unit that is still in place in Bissau, Guinea-Bissau [126]. This trial unit has the capacity to handle 10–20 annual project visits that such trials may entail, as well as provide complex treatments requiring long infusions. The set-up for advanced analyses of immune-mediated processes is in place, both locally and among external partners [65, 71, 73–77, 80, 84, 119, 127–129]. Finally, more collaborations are needed in order to fully take on the many possibilities within this emerging field of HIV-2 cure research, and the Bissau cohorts are open to any collaborative efforts in this area and possible HIV cure applications.

**Table 1:** Strengths and weaknesses of the Bissau HIV and the Guinea-Bissau police cohorts and associated research teams

Strengths	Weaknesses
World’s largest HIV-2 cohort + professional cohort with long and frequent follow-up [4, 12, 98, 130, 131]	High mortality and loss-to-follow-up
HIV-2 epidemiology well-characterized over three decades [4, 22]	
Nationwide cohort [131, 132]	High patient-turnaround and insufficient staff-resources
Strong collaboration with the National Health Laboratory in Guinea-Bissau	Limited lab capacity locally
Large biorepository with preserved plasma and DNA	Limited sample volume in historical samples
Cohort clinical real-time database including demographics and follow-up data [24, 133–135]	Limited data-entry capacity and political instability [136]
Close linkage with HIV-cure research environment and in-depth molecular analysis, including access to humanized mice models, ex vivo infection models, full-length genome sequencing and construction of infectious chimeric viruses [35, 98, 137–141]	Weak local research environment with few nationals at Ph.D level
Well-functioning national ethical committee with enhanced understanding for the complex ethical balancing needed for cure trials	Low health literacy among HIV patients, and extended information and consent procedure needed
National ethics committee placed within Ministry of Health, and a permission also serves as official government authorization for interventions to be tested	Limited experience among official health authorities for approval of non-approved drugs
Burden of co-infections and other comorbidities [98, 136, 142–151]	Limited local diagnostic capacity for a number of co-infections
Resistance testing of HIV-1 [152, 153]	Limited local capacity for genotypic resistance, and non-existing for HIV-2
Well-described algorithms for the diagnostic challenges of differentiating HIV-2 and dual-infections [98, 154–160]	Not the entire cohort tested with updated HIV-2 and HIV-1/HIV-2 dual diagnostics, needs retesting prior to trials

**Table 2:** Challenges with performing an HIV cure trial in Guinea-Bissau and strategies to overcome these

Challenges for HIV cure trials	Strategies to manage challenges
Reluctance to accept high volume blood samples if not sick	Consent for 10 ml EDTA can usually be obtained
Keeping the cold chain	Samples can be transported in cooler to National Health Laboratory and placed in − 80 °C freezer or on dried ice
Cell recovery	Standards for transport of viable PBMCs in place to be analysed elsewhere [18]
Ethical concerns for vulnerable HIV population with limited health literacy [161]	Audio-visual teaching materials can be produced to inform patients relevantly for informed consent
Taboo/stigma of HIV	Staff trained in enrolling and following patients on trials without breaching confidentiality and keeping HIV a secret to other family members in the house
Loss-to-follow-up [98, 162]	Strategies to reduce loss-to-follow-up, including staff trained in mobile phone contact and home visits in place [163]
Considerable adherence challenges, difficult to achieve long-term viral rebound-free treatment [164]	Within the framework of a clinical trial, adherence and follow-up can be improved [126]

## Conclusion

HIV-2 is a less pathogenic virus than HIV-1, disease progression is slower and the proportions of controllers and slow progressors are higher. Both cellular and humoral immune responses, particularly HIV-2-specific CD8+ T-cell responses, are likely to play a role in controlling the rate of disease progression in individuals with dual-infection. A main immunological correlate for the substantial proportion of the aviraemic slow progressors seen in HIV-2 infection is the presence of highly avid, early-differentiated polyfunctional Gag-specific CTL (potentially more effective at targeting reactivated latent virus than HIV-1-specific T-cells). Therefore, there are important lessons to be learned from HIV-2 pathogenesis, and HIV-2 may represent a model to study relapse-free remission in HIV-1 infection (Fig. 1). Understanding the key differences between HIV-1 and HIV-2 infections, and the cross-reactive effects in HIV-1/HIV-2 dual-infection could provide novel insights in developing functional HIV cures and vaccines. There is a clear need to conduct studies on remission-strategies among HIV-2 infected individuals as these studies would provide valuable insights for achieving HIV cure.

## Data Availability

Not applicable.
